# Reversal of ABCG2-mediated MDR by VEGFR3 inhibitor (S)-SAR131675

**DOI:** 10.3389/fphar.2026.1857174

**Published:** 2026-06-18

**Authors:** Xiang Chen, Jiaqian Xu, Ryan Li, Xing-Duo Dong, Yi-Dong Li, Qiu-Xu Teng, Ziqi Wang, Zhe-Sheng Chen

**Affiliations:** 1 Department of Pharmaceutical Sciences, College of Pharmacy and Health Sciences, St. John’s University, Queens, NY, United States; 2 Department of Urology, NHC Key Laboratory of Molecular Probe and Targeted Theragnostic, Harbin Medical University Cancer Hospital, Harbin, China; 3 Perlmutter Cancer Center, NYU Langone Health, New York, NY, United States; 4 Department of Cystoscope Center, Harbin Medical University Cancer Hospital, Harbin, China

**Keywords:** (S)-SAR131675, ABCG2 transporter, multidrug resistance, NSCLC, VEGFR3 inhibitor

## Abstract

Multidrug resistance (MDR) remains a major challenge in cancer treatment, as it is a significant factor contributing to chemotherapy failure and cancer recurrence. Among various resistance mechanisms, the overexpression of the breast cancer resistance protein (BCRP/ABCG2) has been identified as a cause of MDR in multiple cancers, including breast cancer, lung cancer, and colon cancer (S)-SAR131675 is the S-enantiomer of SAR131675 (HY-15458), which significantly and selectively suppresses the Vascular Endothelial Growth Factor Receptor 3 (VEGFR3). In this research (S)-SAR131675 shows the ability to overcome the ABCG2-mediated MDR in both the non-small cell lung cancer cell line NCI-H460 and its ABCG2-overexpressing cell line NCI-H460/TPT10, as well as the colorectal cancer cell line S1 and its ABCG2-overexpressing cell line S1-M1-80. Through several experiments, we demonstrate that (S)-SAR131675 targets the efflux function of ABCG2, resulting in an increased intracellular concentration of substrates of ABCG2, including mitoxantrone and topotecan. Additionally, this resensitizing effect did not affect the overall protein expression or subcellular localization of ABCG2 in the ABCG2-overexpressing cell lines. In summary (S)-SAR131675 can overcome ABCG2-mediated MDR by directly suppressing the drug efflux function, providing a promising basis for the further development of cancer treatments associated with MDR in the future.

## 1 Introduction

Over the past several decades, cancer has remained a major global health challenge. Despite extensive research and the emergence of various innovative therapies, multidrug resistance (MDR) is widely recognized as the foremost factor contributing to treatment failure or cancer recurrence (Ge et al., 2025). Both inherent properties and acquired resistance during treatment could lead to MDR, but ultimately, both result in treatment failure (Wang et al., 2021; Teng et al., 2024). The ATP-binding cassette (ABC) transporter superfamily has been demonstrated to actively mediate the transmembrane transport of various endogenous and exogenous molecules by hydrolyzing ATP as an energy source (Wang et al., 2021), which plays a central role among the mechanisms of MDR. The ABC transporter superfamily comprises seven subfamilies, designated from ABCA to ABCG, with a total of 49 membrane transporters currently identified (Ge et al., 2025; Wang et al., 2021; Yu et al., 2025). Within this superfamily, ABCG2 (Breast Cancer Resistance Protein, BCRP) is a pivotal mediator of resistance to a broad spectrum of substrates, including mitoxantrone, topotecan, and methotrexate, by reducing intracellular drug accumulation (Allikmets et al., 1998; Chen et al., 2024). Subsequent studies expanded the range of ABCG2 substrates to include camptothecin analogs, such as irinotecan and SN-38, flavopiridol, and several tyrosine kinase inhibitors, reflecting its broad substrate spectrum and clinical relevance (Noguchi et al., 2014; Giri et al., 2009; Narayanan et al., 2022; Zhao et al., 2025).

To counteract ABCG2-mediated MDR, several generations of inhibitors have been developed. However, their clinical benefits are significantly impeded by dose-limiting toxicities, unfavorable pharmacokinetic interactions with chemotherapeutics, and insufficient selectivity (Szakács et al., 2006; Robey et al., 2018). Recently, several new targets have been developed, including tyrosine kinase inhibitors and natural and synthetic compounds such as curcumin and resveratrol, which show potential to overcome ABCG2-mediated MDR by regulating protein expression and function, with improved tolerability (Nabekura, 2010; Kukal et al., 2021). Despite ongoing challenges in efficacy, safety, and patient stratification, combination therapy strategies integrating ABCG2 inhibitors with standard chemotherapy agents remain under active investigation and have yielded encouraging preclinical findings (Robey et al., 2018; He and Wei, 2012). Vascular endothelial growth factor receptor-3 (VEGFR3), also known as FLT4, is a receptor tyrosine kinase of the VEGFR family that plays a central role in regulating lymphangiogenesis, vascular remodeling, endothelial survival, and cell migration (Tammela and Alitalo, 2010; Alitalo, 2011). Signaling cascades downstream of VEGFR3, such as PI3K/AKT, MAPK/ERK, and PLCγ pathways, are known to modulate the expression and membrane localization of ABCG2 (Alitalo, 2011; Koch and Claesson-Welsh, 2012; Lin et al., 2023; Zhang et al., 2020; Zhang et al., 2022; De Vera et al., 2019). Accumulating evidence indicates that several VEGFR inhibitors can modulate ABC transporters (Szakács et al., 2006). Importantly, multiple VEGFR inhibitors, including sorafenib, sunitinib, apatinib, and regorafenib, have been reported to reverse ABC transporter-mediated MDR by inhibiting transporter function and increasing intracellular accumulation of substrate anticancer drugs (Szakács et al., 2006; Hu et al., 2009; Mi et al., 2010).

Furthermore, as a selective small-molecule VEGFR-3 inhibitor (S)-SAR131675 exhibits sub-nanomolar inhibitory activity against VEGFR3 with high specificity over VEGFR1, VEGFR2, and other kinases. While SAR131675 has been demonstrated in previous studies to block angiogenesis, lymphangiogenesis, and TAM infiltration across various experimental cancer models (Alam et al., 2012). The use of a single (S)-enantiomer may reduce variability associated with stereoisomeric mixtures and enable a more consistent evaluation of its biological effects. Therefore (S)-SAR131675 was used throughout the present study instead of the racemic mixture.

In this study, we aim to evaluate the potential of (S)-SAR131675 to modulate ABCG2-mediated MDR in cancer cells. We utilized various *in vitro* models to investigate its effect on the sensitivity of ABCG2-overexpressing cells to substrate chemotherapeutics. Furthermore, we conducted mechanistic studies, including drug accumulation assays and Western blot analysis, to determine whether (S)-SAR131675 reverses MDR by inhibiting the transport activity of ABCG2 or by modulating its protein expression.

## 2 Materials and methods

### 2.1 Chemical

Dulbecco’s modified Eagle’s medium (DMEM), fetal bovine serum, and penicillin–streptomycin were obtained from HyClone (Logan, UT, United States). Alexa Fluor 488–labeled secondary IgG and a GAPDH monoclonal antibody (clone GA1R, MA5-15738) were purchased from Thermo Fisher Scientific (Rockford, IL, United States). The monoclonal antibody against ABCG2 (clone BXP-21, MAB4146) was supplied by Millipore (Billerica, MA, United States). An HRP-conjugated anti-mouse IgG secondary antibody (7076S) was acquired from Cell Signaling Technology (Danvers, MA, United States). Cisplatin, mitoxantrone, MTT, DMSO, DAPI, and Triton X-100 were sourced from Sigma-Aldrich (St. Louis, MO, United States). Ko143 and geneticin (G418) were purchased from Enzo Life Sciences (Farmingdale, NY, United States). Phosphate-buffered saline (PBS) and bovine serum albumin (BSA) were obtained from VWR Chemicals (Solon, OH, United States) [^3^H]-mitoxantrone (2.5 Ci/mmol) was supplied by Moravek Biochemicals (Brea, CA, United States), and the liquid scintillation cocktail was purchased from MP Biomedicals (Santa Ana, CA, United States). Topotecan (TPT) was obtained from Chemietek (Indianapolis, IN, United States), and (S)-SAR131675 was purchased from MedChemExpress (Monmouth Junction, NJ, United States). Unless otherwise specified, all other reagents were obtained from Sigma-Aldrich (St. Louis, MO, United States).

### 2.2 Cell lines and cell culture

The human non–small cell lung cancer (NSCLC) cell line NCI-H460 and colorectal cancer cell line S1 were maintained in DMEM supplemented with 10% fetal bovine serum and 1% penicillin/streptomycin. These cell lines were kindly provided by Dr. Susan E. Bates (Columbia University, New York, NY, United States) and Dr. Robert Robey (NIH, Bethesda, MD, United States). The topotecan-resistant, ABCG2-overexpressing subline NCI-H460/TPT10 was cultured under identical conditions with 10 μM topotecan, and cells were transferred to drug-free medium for at least 2 weeks prior to experimentation (Lei et al., 2020a). An ABCG2 knockout derivative of NCI-H460/TPT10 was generated using the CRISPR/Cas9 system and continuously maintained in medium containing 1.5 mg/mL G418 (Lei et al., 2020a; Zhang et al., 2018). Human embryonic kidney cell models included HEK293/pcDNA3.1, HEK293/ABCG2-482-R2, HEK293/ABCG2-482-G2, and HEK293/ABCG2-482-T7. HEK293/pcDNA3.1 cells were transfected with the empty pcDNA3.1 vector, whereas HEK293/ABCG2-482-R2/G2/T7 cells were transfected with a pcDNA3.1 construct encoding full-length ABCG2, where R2, G2, and T7 indicate different amino acid substitutions (Arginine, Glycine, or Threonine) at position 482, and these cells were selected and cultured in DMEM with 2 mg/mL G418 (Zhang et al., 2018). The human epidermoid carcinoma cell line KB-three to one and its multidrug-resistant sublines KB-C2 and KB-CV60, which overexpress ABCB1 and ABCC1, respectively, were cultured in DMEM supplemented with 10% fetal bovine serum and 1% penicillin/streptomycin. Resistant sublines were maintained in medium containing 2 μg/mL colchicine (KB-C2) or 1 μg/mL of Cepharanthine and 60 ng/mL of vincristine (KB-CV60). All cell lines were incubated at 37 °C in a humidified atmosphere with 5% CO_2_.

### 2.3 Cell viability assay

Cell viability and reversal effects were evaluated using the MTT colorimetric assay, as previously described (Zhang et al., 2022; Dong et al., 2024a). Briefly, depending on the growth characteristics of each cell line, 5,000–10,000 cells per well were seeded into 96-well plates in 160 μL of complete culture medium and allowed to adhere overnight at 37 °C in a humidified incubator with 5% CO_2_. Cells were then exposed to various concentrations of test compounds. For reversal experiments, the reversal agents were added 4 h prior to the addition of substrate drugs. Both reversal agents and substrate drugs were prepared in culture medium and added at 20 μL per well. Following a total incubation period of 68 h, 20 μL of MTT solution (4 mg/mL) was added to each well, and the plates were incubated for an additional 4 h. The culture medium was subsequently removed, and the resulting formazan crystals were dissolved in 100 μL of DMSO. Absorbance was measured at 570 nm using a microplate reader. Cell viability was expressed as a percentage of untreated controls, and IC_50_ values were calculated from dose–response curves generated using nonlinear regression analysis. Except for cisplatin, which was dissolved in dimethylformamide (DMF), all tested compounds were dissolved in dimethyl sulfoxide (DMSO) to prepare 10 mM stock solutions. In all experiments, the final DMSO concentration did not exceed 0.1% (v/v).

### 2.4 Western blot analysis

Western blot analysis was performed to examine protein expression levels as previously described (Yang et al., 2025; Cao et al., 2026). Briefly, NCI-H460/TPT10 cells were treated with (S)-SAR131675 at different concentrations (1, 3, 5 μM for 72 h) or at 5 μM for various time points (0, 24, and 48 h). Following treatment, cells were washed with cold PBS and lysed on ice for 20 min using lysis buffer containing 25 mM Tris, 0.15% EDTA, 1% sodium deoxycholate, 0.1% SDS, 0.88% NaCl, 1% Triton X-100, and a protease inhibitor cocktail. Lysates were then centrifuged at 12,000 rpm for 20 min at 4 °C, and the supernatants were collected as total protein extracts. Protein concentrations were determined using a bicinchoninic acid (BCA) protein assay kit (Thermo Scientific, Rockford, IL, United States). Equal amounts of protein were subsequently separated by SDS–polyacrylamide gel electrophoresis (SDS-PAGE) and transferred onto polyvinylidene fluoride (PVDF) membranes. Membranes were blocked with 5% non-fat dry milk in TBST for 2 h at room temperature and then incubated overnight at 4 °C with primary antibodies against ABCG2 (BXP-34) and GAPDH (GA1R). After washing with TBST, membranes were incubated with HRP-conjugated anti-mouse IgG secondary antibody for 2 h at room temperature. Protein bands were visualized using an enhanced chemiluminescence (ECL) detection system. Densitometric analysis was performed using ImageJ software (NIH, Bethesda, MD, United States), and protein expression levels were normalized to GAPDH.

### 2.5 Immunofluorescence assay

Protein expression and localization were examined by immunofluorescence, as previously described (Dong et al., 2024b). Immunofluorescence assays were conducted in 24-well plates designed for this assay by seeding 10,000 NCI-H460 and NCI-H460/TPT10 cells per well. After overnight incubation, the cells were exposed to different concentrations of (S)-SAR131675 for different times, including 1 and 5 μM for 72 h or 5 μM for 0, 24, and 48 h. Then, the cells were fixed with 4% paraformaldehyde for 10 min, permeabilized with 0.1% Triton X-100 for 10 min, and blocked with 6% BSA for 1 h; all reagents were diluted in PBS. Between steps, during the process, the cells were washed 3 times with cold PBS. Then, the blocking buffer was collected and used to prepare the primary monoclonal antibody against ABCG2 (1:1000) and Alexa Fluor 488-conjugated IgG secondary antibody (1:1000). Cells were incubated with the primary monoclonal antibody overnight at 4 °C. Afterward, the plate was moved to a dark environment, and the secondary antibody was added to the cells for 2 h at room temperature, followed by three washes with cold PBS. The DAPI solution was then added, and the cells were incubated in the dark at 37 °C for 30 min. After a final wash in cold PBS, images were captured using an EVOS FL Auto fluorescence microscope (Life Technologies Corporation, Gaithersburg, MD), and ImageJ software (NIH, Bethesda, MD, United States) was used to examine and quantify fluorescence intensity and assess subcellular localization.

### 2.6 ATPase assay

The vanadate-sensitive ATPase activity assay was performed using membrane vesicles prepared from High Five insect cells, as previously described (Yang et al., 2025; Dong et al., 2020). Briefly, membrane vesicles (10 μg of protein) were incubated at 37 °C in ATPase assay buffer in the presence or absence of 0.3 mmol/L sodium orthovanadate. The assay buffer consisted of 50 mM MES (pH 6.8), 50 mmol/L KCl, 5 mM sodium azide, 2 mM EGTA, 2 mM DTT, 1 mM ouabain, and 10 mM MgCl_2_. Membrane vesicles were pre-incubated with (S)-SAR131675 (0–20 μM) for 3 min at 37 °C. The ATPase reaction was initiated by adding 5 mM Mg-ATP (100 μL) and incubated for 20 min at 37 °C. The reaction was terminated by the addition of an equal volume of 5% SDS solution. The amount of inorganic phosphate (Pi) released was determined spectrophotometrically at 800 nm, and vanadate-sensitive ATPase activity was calculated as the difference between total ATPase activity and the activity measured in the presence of sodium orthovanadate, representing ABCG2-specific ATPase activity.

### 2.7 [^3^H]-mitoxantrone accumulation assay

The intracellular accumulation assay was performed using NCI-H460 cells and NCI-H460/TPT10 cells, as previously described (Lei et al., 2020b). Briefly, cells were seeded at 5 × 10^4^ cells per well in 24-well plates and incubated overnight. Cells were pretreated with 5 μM (S)-SAR131675 for 2 h, followed by the addition of 20 nM [^3^H]-mitoxantrone. The treated cells were then incubated for an additional 2 h in the presence or absence of (S)-SAR131675. After incubation, the cells were washed twice with ice-cold PBS, trypsinized, and collected. Cell suspensions were transferred into scintillation vials containing 5 mL of scintillation fluid, and intracellular radioactivity was measured using a Packard TRI-CARB 1900CA liquid scintillation analyzer (Packard Instrument, Downers Grove, IL, United States). Accumulation of [^3^H]-mitoxantrone was expressed as counts per minute (CPM) and normalized to cell number or protein content.

### 2.8 [^3^H]-mitoxantrone efflux assay

The efflux assay was performed using NCI-H460 and NCI-H460/TPT10 cells, as previously described (Teng et al., 2024). The initial procedures were identical to those of the accumulation assay up to the point of washing the cells twice with ice-cold PBS. After [^3^H]-mitoxantrone loading, cells were incubated in drug-free medium with or without (S)-SAR131675 to allow efflux to proceed. At designated time points (0, 30, 60, and 120 min), cells were trypsinized and collected. Cell suspensions were transferred into scintillation vials containing 5 mL of scintillation fluid, and intracellular radioactivity was quantified using a Packard TRI-CARB 1900CA liquid scintillation analyzer (Packard Instrument, Downers Grove, IL, United States). The rate of drug efflux was expressed as the percentage of intracellular [^3^H]-mitoxantrone remaining relative to that measured at time zero.

### 2.9 Molecular modeling of human ABCG2 and docking of (S)-SAR131675

Molecular docking analysis was performed using Maestro 11.5 (Schrödinger, LLC, New York, NY, United States, 2018) as previously described (Dong et al., 2024a; Dong et al., 2020). Ligand and protein preparations were performed using the standard LigPrep and Protein Preparation Wizard workflows, respectively. The receptor grid was generated based on the substrate-binding pocket of human ABCG2 (PDB ID: 5NJ3), with key residues Phe432, Phe439, Leu539, Ile543, Val546, and Met549 selected to define the docking site (Zhang et al., 2018). Initial molecular docking was performed using the Glide extra precision (XP) mode. The top-ranked ligand–protein complex from the Glide XP docking was then used to generate the receptor grid for induced-fit docking (IFD). Subsequently, IFD was performed using the default Schrödinger protocol to enable flexible refinement of both the ligand conformation and receptor side chains. The final docking poses were selected based on docking scores and a visual inspection of the binding interactions.

### 2.10 Statistical analysis

All data are presented as mean ± standard deviation (SD). Statistical analyses were performed using GraphPad Prism 7.0 (GraphPad Software, San Diego, CA, United States). Comparisons among multiple groups were conducted using one-way analysis of variance (ANOVA), except for the efflux experiments, which were analyzed using two-way ANOVA. Afterward, the *post hoc* Tukey’s test was conducted to further evaluate differences among groups. All experiments were repeated at least three times. Statistically significant levels were defined as *p < 0.05; **p < 0.01; ***p < 0.001; ****p < 0.0001.

## 3 Results

### 3.1 (S)-SAR131675 overcomes the ABCG2-mediated MDR in ABCG2-overexpressing cell lines

**FIGURE 1:**
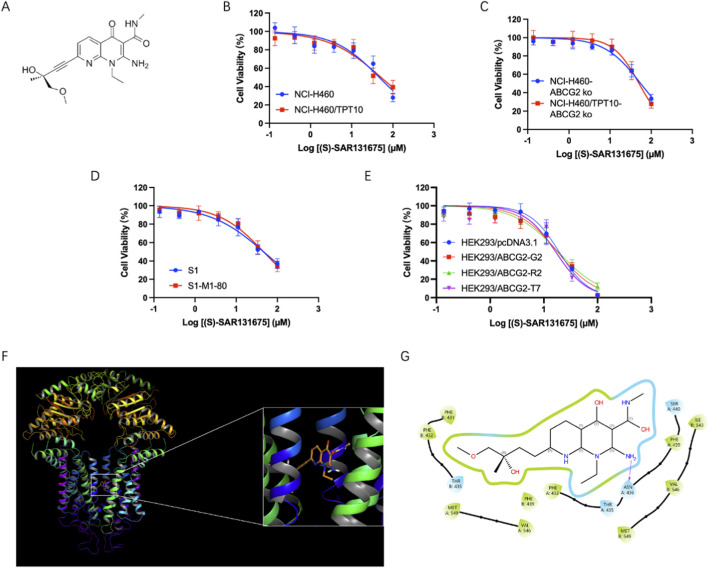
**(A)** Chemical structure of (S)-SAR131675. **(B)** Cytotoxicity results of (S)-SAR131675 in NCI-H460 and its ABCG2-Overexpressing cell line NCI-H460/TPT10. **(C)** Cytotoxicity results of (S)-SAR131675 in ABCG2-knockout cell line NCI-H460-ABCG2 ko and NCI-H460/TPT10-ABCG2 ko. **(D)** Cytotoxicity results of (S)-SAR131675 in S1 and its ABCG2-Overexpressing cell line S1-M1-80. **(E)** Cytotoxicity results of (S)-SAR131675 in HEK293/pcDNA3.1 and its ABCG2-overexpressing cell line HEK293/ABCG2-482-R2, HEK293/ABCG2-482-G2, HEK293/ABCG2-482-T7. **(F)** Docked position of (S)-SAR131675 within the drug-binding site of ABCG2. **(G)** The strong binding of ligands within the ABCG2 pocket, docking analysis of the binding of (S)-SAR131675 with the human ABCG2 homology model cryo-EM structure (PDB ID: 5NJ3).

Firstly, we investigated whether (S)-SAR131675 is a substrate of ABCG2. As shown in Figures 1B–E and Table 1, the IC_50_ values of (S)-SAR131675 did not differ significantly between parental and ABCG2-overexpressing resistant cell lines, indicating that (S)-SAR131675 is not a substrate of ABCG2. Furthermore, to perform the reversal experiments appropriately, it was necessary to establish a non-toxic concentration of (S)-SAR131675 that does not affect cell survival. Based on the MTT assay description, the IC_20_ was used as the threshold for non-toxic exposure after 72 h of incubation. Based on Figures 1B–E and Table 1, we determined the IC_20_ of (S)-SAR131675 and decided to use 5 μM, 3 μM, and 1 μM in the reversal experiments for all cell lines, including NCI-H460, NCI-H460/TPT10, and its knockout cell lines, S1, S1-M1-80, HEK293/pcDNA3.1, HEK293/ABCG2-482-R2, HEK293/ABCG2-482-G2, and HEK293/ABCG2-482-T7. Among these cell lines, NCI-H460/TPT10 is defined as a wild-type ABCG2-overexpressing cell line, and S1-M1-80 is defined as a mutant-type ABCG2-overexpressing cell line harboring a glycine-for-arginine substitution at position 482. For the HEK293 cell lines, HEK293/pcDNA3.1 serves as the parental cell line lacking ABCG2 expression. HEK293/ABCG2-482-R2 is the wild-type ABCG2-overexpressing cell line, whereas HEK293/ABCG2-482-G2 and HEK293/ABCG2-482-T7 are mutant-type ABCG2-overexpressing cell lines that harbor a threonine (T7) or glycine (G2) substitution for arginine (R2) at position 482.

**TABLE 1:** IC_50_ values of the (S)-SAR131675 cytotoxicity experiments.

Cell lines	IC_50_ ± SD (μM) (R_R_)
NCI-H460	46.67 ± 9.462 (1.00)
NCI-H460/TPT10	51.52 ± 6.784 (0.91)
NCI-H460 ko	55.41 ± 7.572 (1.00)
NCI-H460/TPT10 ko	50.50 ± 9.972 (1.10)
S1	44.82 ± 11.107 (1.00)
S1-M1-80	56.95 ± 9.972 (0.79)
HEK293/pcDNA3.1	19.18 ± 2.591 (1.00)
HEK293/ABCG2-482-R2	17.76 ± 2.097 (1.08)
HEK293/ABCG2-482-G2	18.48 ± 1.158 (1.04)
HEK293/ABCG2-482-T7	16.52 ± 3.556 (1.16)

Data are presented as mean ± SD from three independent experiments. The resistance reversal ratio (R_R,_ defined as IC_50_ parental cell line/IC_50_ resistance cell line) was calculated. R_R_ values are shown in parentheses. Statistical significance was determined by comparison with the single chemotherapeutic-treated group in each cell line: *p < 0.05; **p < 0.01; ***p < 0.001; ****p < 0.0001.

**FIGURE 2:**
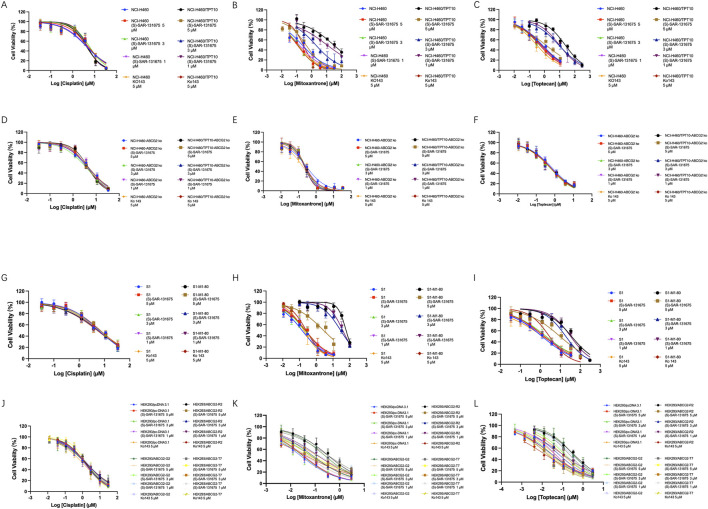
**(A-C),** Cytotoxicity of Cisplatin, Mitoxantrone, and Topotecan in NCI-H460 cells and the ABCG2-overexpressing cell line NCI-H460/TPT10 following treatment with chemotherapeutic drug alone or in combination with different concentrations of (S)-SAR131675 or Ko143. **(D-F)**, Cytotoxicity of Cisplatin, Mitoxantrone, and Topotecan in ABCG2-knockout cell lines, NCI-H460-ABCG2 ko and NCI-H460/TPT10-ABCG2 ko, following treatment with chemotherapeutic drug alone or in combination with different concentrations of (S)-SAR131675 or Ko143. **(G-I)**, Cytotoxicity of Cisplatin, Mitoxantrone, and Topotecan in S1 and its ABCG2-overexpressing cell line S1-M1-80, following treatment with chemotherapeutic drug alone or in combination with different concentrations of (S)-SAR131675 or Ko143. **(J-L),** Cytotoxicity of Cisplatin, Mitoxantrone, and Topotecan in HEK293/pcDNA3.1 and its ABCG2-overexpressing cell lines, HEK293/ABCG2-482-R2, HEK293/ABCG2-482-G2, HEK293/ABCG2-482-T7, following treatment with chemotherapeutic drug alone or in combination with different concentrations of (S)-SAR131675 or Ko143. Data are presented as mean ± SD from three independent experiments. *p < 0.05; **p < 0.01; ***p < 0.001; ****p < 0.0001.

Figures 2B,C show that (S)-SAR131675 significantly resensitized the ABCG2-overexpressing cell lines to ABCG2 substrate drugs, including mitoxantrone and topotecan, in a concentration-dependent manner. Additionally, compared to Ko143, a known positive ABCG2 reversal agent (S)-SAR131675 showed a less potent reversal effect at the same concentration. In Figure 2A, cisplatin, a non-ABCG2 substrate used as a negative control, did not exhibit any reversal effect with or without (S)-SAR131675 or Ko143, confirming the ABCG2-specific nature of the sensitization. Furthermore, in Figures 2D-F, the reversal experiments in NCI-H460-ABCG2 ko and NCI-H460/TPT10-ABCG2 ko cells demonstrated that the reversal effect of (S)-SAR131675 is ABCG2-dependent. Regardless of whether mitoxantrone, topotecan, or cisplatin was used, no significant changes in IC_50_ values were observed following treatment with Ko143 or (S)-SAR131675, indicating that in the absence of ABCG2 overexpression (S)-SAR131675 did not alter drug sensitivity.

The reversal effect was further evaluated in the S1 cell line and its ABCG2-overexpressing cell line, S1-M1-80. Figures 2H,I show that (S)-SAR131675 significantly resensitized S1-M1-80 cells to both mitoxantrone and topotecan, similar to NCI-H460/TPT10, although it exhibited a less potent reversal effect compared to Ko143. As shown in Figure 2G, neither Ko143 nor (S)-SAR131675 altered the IC_50_ value of cisplatin, further confirming the ABCG2-specific nature of the reversal effect.

Reversal experiments were also performed in the HEK293 cell lines, including HEK293/pcDNA3.1, HEK293/ABCG2-482-R2, HEK293/ABCG2-482-G2, and HEK293/ABCG2-482-T7. As shown in Figures 2K,L, (S)-SAR131675 exhibited a comparable reversal effect across HEK293/ABCG2 cell lines, significantly resensitizing the cells to both mitoxantrone and topotecan; however, at the same concentration, its potency was lower than that of Ko143. As shown in Figure 2J, no significant change in the IC_50_ of cisplatin was observed with or without Ko143 or (S)-SAR131675. These results further confirm that the reversal effect of (S)-SAR131675 is ABCG2-specific. All the figures in Figure 2 present data as the mean ± SD of three independent experiments. According to Tables 2, 3 the IC_50_ values are the mean ± SD from three independent experiments. Then, the KB-3-1 and its ABCB1-overexpressing cell line KB-C2, and the ABCC1-overexpressing cell line KB-CV60 were used to confirm ABCG2 specificity, which was previously shown to have high ABCB1 and ABCC1 protein expression levels (Chen et al., 2021; Cao et al., 2026). Tables 4, 5; Figure 5, our findings suggest that its reversal activity may also exhibit a higher degree of transporter selectivity toward ABCG2, as no significant effects were observed in ABCB1- or ABCC1-overexpressing cell models.

**TABLE 2:** IC_50_ values of Cisplatin, Mitoxantrone, and Topotecan in the presence or absence of (S)-SAR131675 or Ko143.

IC_50_ ± SD (μM) (R_R_)						
Cell line treatment	NCI-H460	NCI-H460/TPT10	NCI-H460-ABCG2 ko	NCI-H460/TPT10-ABCG2 ko	S1	S1-M1-80
Mitoxantrone	0.2318 ± 0.1457 (1.00)	29.3600 ± 2.411 (1.00)	0.2741 ± 0.0489 (1.00)	0.2358 ± 0.0763 (1.00)	0.1382 ± 0.0318 (1.00)	60.3200 ± 2.5403 (1.00)
+Ko143 3 μM	0.1255 ± 0.0369 (1.85)	0.2754 ± 0.0426 (106.61) ****	0.1716 ± 0.0004 (1.60)	0.2303 ± 0.0472 (1.02)	0.1768 ± 0.0501 (0.78)	0.2468 ± 0.1178 (244.41) ****
+(S)-SAR131675 5 μM	0.1073 ± 0.0172 (2.16)	0.4149 ± 0.1230 (70.76) ****	0.2344 ± 0.0453 (1.17)	0.2530 ± 0.0358 (0.93)	0.2260 ± 0.1285 (0.61)	2.6700 ± 0.4491 (22.59) ****
+(S)-SAR131675 3 μM	0.1166 ± 0.0200 (1.99)	1.8700 ± 0.3426 (15.70) ****	0.2392 ± 0.0382 (1.15)	0.2203 ± 0.0705 (1.07)	0.2054 ± 0.1685 (0.67)	28.2100 ± 5.1651 (2.14) ****
+(S)-SAR131675 1 μM	0.1460 ± 0.0763 (1.59)	10.5300 ± 0.54 (2.79) ****	0.2462 ± 0.0591 (1.11)	0.2138 ± 0.0433 (1.10)	0.2133 ± 0.0842 (0.65)	37.8800 ± 9.7183 (1.59) ***
Topotecan	0.7092 ± 0.1324 (1.00)	23.0000 ± 3.6514 (1.00)	0.7468 ± 0.0249 (1.00)	0.6332 ± 0.0507 (1.00)	1.2010 ± 0.0201 (1.00)	36.0500 ± 8.3476 (1.00)
+Ko143 3 μM	0.3070 ± 0.0753 (2.31)	0.6140 ± 0.1843 (37.46) ****	0.5621 ± 0.1161 (1.33)	0.6092 ± 0.1855 (1.04)	1.3490 ± 0.0498 (0.89)	2.8110 ± 0.6283 (12.82) ****
+(S)-SAR131675 5 μM	0.4801 ± 0.0527 (1.48)	1.2010 ± 0.3837 (19.15) ****	0.6176 ± 0.0742 (1.21)	0.6323 ± 0.0977 (1.00)	1.5550 ± 0.4460 (0.77)	9.5210 ± 1.4503 (3.79) ***
+(S)-SAR131675 3 μM	0.4623 ± 0.0843 (1.53)	8.5920 ± 2.4176 (2.68) ****	0.5721 ± 0.1052 (1.31)	0.6652 ± 0.1908 (0.95)	1.9170 ± 0.3736 (0.63)	18.6700 ± 3.6667 (1.93) **
+(S)-SAR131675 1 μM	0.5256 ± 0.1368 (1.35)	9.4670 ± 2.6576 (2.43) ***	0.6479 ± 0.1490 (1.15)	0.6696 ± 0.1368 (0.95)	1.5740 ± 0.5437 (0.76)	32.4700 ± 5.6785 (1.11)
Cisplatin	4.031 ± 0.6075 (1.00)	4.211 ± 0.8071 (1.00)	4.760 ± 0.6841 (1.00)	4.606 ± 0.6120 (1.00)	6.159 ± 1.2186 (1.00)	5.481 ± 0.9308 (1.00)
+Ko143 3 μM	4.093 ± 0.2705 (0.98)	4.624 ± 0.7018 (0.91)	4.985 ± 1.0186 (1.05)	4.879 ± 0.2056 (0.95)	5.962 ± 1.1600 (1.03)	5.035 ± 0.5866 (1.09)
+(S)-SAR131675 5 μM	4.854 ± 0.4550 (0.83)	4.031 ± 0.4227 (1.04)	4.804 ± 0.5952 (1.01)	4.223 ± 0.5008 (0.99)	7.000 ± 2.0741 (0.88)	6.667 ± 0.8159 (0.82)
+(S)-SAR131675 3 μM	5.591 ± 1.1409 (0.72)	4.510 ± 0.7450 (0.93)	3.825 ± 0.2512 (0.80)	4.103 ± 0.7800 (1.24)	6.672 ± 1.9221 (0.92)	5.516 ± 1.0465 (0.99)
+(S)-SAR131675 1 μM	4.317 ± 1.3035 (0.93)	4.488 ± 1.0687 (0.94)	4.822 ± 0.8916 (1.01)	4.252 ± 0.5246 (0.99)	5.851 ± 0.9733 (1.05)	5.538 ± 0.5932 (0.99)

Data are presented as mean ± SD from three independent experiments. The resistance reversal ratio (R_R;_ defined as IC_50_ chemotherapeutic drug alone/IC_50_ combination) was calculated for each drug combination in each cell line. R_R_ values are shown in parentheses. Statistical significance was determined by comparison with the single chemotherapeutic-treated group in each cell line: *p < 0.05; **p < 0.01; ***p < 0.001; ****p < 0.0001.

**TABLE 3:** IC_50_ values of Cisplatin, Mitoxantrone, and Topotecan in the presence or absence of (S)-SAR131675 or Ko143.

IC_50_ ± SD (μM) (R_R_)				
Cell line treatment	HEK293/pcDNA3.1	HEK293/ABCG2-482-R2	HEK293/ABCG2-482-G2	HEK293/ABCG2-482-T7
Mitoxantrone	0.0237 ± 0.0019 (1.00)	0.3231 ± 0.0470 (1.00)	0.2329 ± 0.0439 (1.00)	0.3193 ± 0.0732 (1.00)
+Ko143 3 μM	0.0292 ± 0.0080 (0.81)	0.0367 ± 0.0060 (8.80) ****	0.0368 ± 0.0089 (6.33) ****	0.0478 ± 0.0084 (6.68) ****
+(S)-SAR131675 5 μM	0.0302 ± 0.0060 (0.78)	0.0597 ± 0.0108 (5.41) ****	0.0373 ± 0.0050 (6.24) ****	0.0727 ± 0.0142 (4.39) ****
+(S)-SAR131675 3 μM	0.0283 ± 0.0148 (0.84)	0.0736 ± 0.0163 (4.39) ****	0.0628 ± 0.0200 (3.71) ****	0.1111 ± 0.0166 (2.87) ***
+(S)-SAR131675 1 μM	0.0319 ± 0.0078 (0.74)	0.1200 ± 0.0109 (2.69) ****	0.1322 ± 0.0238 (1.76) ***	0.2590 ± 0.0388 (1.23)
Topotecan	0.0272 ± 0.0053 (1.00)	0.3274 ± 0.0454 (1.00)	0.3073 ± 0.0336 (1.00)	0.2006 ± 0.0226 (1.00)
+Ko143 3 μM	0.0393 ± 0.0033 (0.69)	0.0125 ± 0.0010 (26.19) ****	0.0213 ± 0.0053 (14.43) ****	0.0176 ± 0.0039 (11.40) ****
+(S)-SAR131675 5 μM	0.0277 ± 0.0063 (0.98)	0.0171 ± 0.0026 (19.15) ****	0.0236 ± 0.0061 (13.02) ****	0.0203 ± 0.0037 (9.88) ****
+(S)-SAR131675 3 μM	0.0282 ± 0.0058 (0.96)	0.0605 ± 0.0127 (5.41) ****	0.1204 ± 0.0171 (2.55) ****	0.0911 ± 0.0096 (2.20) ****
+(S)-SAR131675 1 μM	0.0415 ± 0.0028 (0.66)	0.0923 ± 0.0149 (3.55) ****	0.2575 ± 0.0486 (1.19)	0.1422 ± 0.0089 (1.41) ***
Cisplatin	1.495 ± 0.0785 (1.00)	1.718 ± 0.2019 (1.00)	1.765 ± 0.1760 (1.00)	1.777 ± 0.3837 (1.00)
+Ko143 3 μM	1.167 ± 0.1441 (1.28)	1.901 ± 0.3128 (0.90)	2.258 ± 0.1900 (0.78)	1.636 ± 0.3675 (1.09)
+(S)-SAR131675 5 μM	1.634 ± 0.2180 (0.91)	1.574 ± 0.4046 (1.09)	1.787 ± 0.1345 (0.99)	1.863 ± 0.1951 (0.95)
+(S)-SAR131675 3 μM	1.585 ± 0.1955 (0.94)	1.991 ± 0.1374 (0.86)	1.646 ± 0.2991 (1.07)	1.504 ± 0.3446 (1.18)
+(S)-SAR131675 1 μM	1.435 ± 0.1789 (1.04)	1.830 ± 0.1264 (0.94)	1.678 ± 0.1811 (1.05)	1.500 ± 0.2163 (1.18)

Data are presented as the mean ± SD from three independent experiments. The resistance reversal ratio (R_R;_ defined as IC_50_ chemotherapeutic drug alone/IC_50_ combination) was calculated for each drug combination in each cell line. R_R_ values are shown in parentheses. Statistical significance was determined by comparison with the single chemotherapeutic-treated group in each cell line: *p < 0.05; **p < 0.01; ***p < 0.001; ****p < 0.0001.

**TABLE 4:** IC_50_ values of Paclitaxel in the presence or absence of (S)-SAR131675 or Verapamil.

IC_50_ ± SD (μM) (RR)		
Cell line treatment	KB-3-1	KB-C2
Paclitaxel	0.0176 ± 0.0018 (1.00)	1.0900 ± 0.1091 (1.00)
+Verapamil 5 μM	0.0190 ± 0.0138 (0.93)	0.0094 ± 0.0026 (115.96) **
+(S)-SAR131675 5 μM	0.0170 ± 0.0009 (1.04)	1.2400 ± 0.0745 (0.88)
+(S)-SAR131675 3 μM	0.0211 ± 0.0054 (0.83)	1.3680 ± 0.5982 (0.80)
+(S)-SAR131675 1 μM	0.0212 ± 0.0065 (0.83)	0.9829 ± 0.1862 (1.11)

Data are presented as the mean ± SD from three independent experiments. The resistance reversal ratio (R_R;_ defined as IC_50_ chemotherapeutic drug alone/IC_50_ combination) was calculated for each drug combination in each cell line. R_R_ values are shown in parentheses. Statistical significance was determined by comparison with the single chemotherapeutic-treated group in each cell line: *p < 0.05; **p < 0.01; ***p < 0.001; ****p < 0.0001.

**TABLE 5:** IC_50_ values of Paclitaxel in the presence or absence of (S)-SAR131675 or MK571.

IC_50_ ± SD (μM) (RR)		
Cell line treatment	KB-3-1	KB-CV60
Paclitaxel	0.6926 ± 0.1861 (1.00)	14.16 ± 2.7000 (1.00)
+MK571 25 μM	0.5167 ± 0.1588 (1.34)	1.43 ± 0.2459 (9.90) **
+(S)-SAR131675 5 μM	0.6157 ± 0.2365 (1.12)	13.07 ± 2.9846 (1.08)
+(S)-SAR131675 3 μM	0.6334 ± 0.1737 (1.09)	15.04 ± 4.0413 (0.94)
+(S)-SAR131675 1 μM	0.5124 ± 0.1940 (1.35)	15.15 ± 3.4626 (0.93)

Data are presented as the mean ± SD from three independent experiments. The resistance reversal ratio (R_R;_ defined as IC_50_ chemotherapeutic drug alone/IC_50_ combination) was calculated for each drug combination in each cell line. R_R_ values are shown in parentheses. Statistical significance was determined by comparison with the single chemotherapeutic-treated group in each cell line: *p < 0.05; **p < 0.01; ***p < 0.001; ****p < 0.0001.

### 3.2 (S)-SAR131675 did not significantly alter the expression level of ABCG2 and subcellular localization in ABCG2-overexpressing cancer cells

Based on the MTT assay results (S)-SAR131675 was identified as a potent modulator that effectively reverses ABCG2-mediated MDR. Elucidation of the molecular mechanisms underlying this reversal effect is critical for advancing the understanding of ABCG2-mediated MDR and for guiding future drug development. Currently, several mechanisms have been recognized in previous studies, including alteration of ABCG2 protein expression and subcellular localization. To evaluate these possibilities, Western blotting and immunofluorescence assays were performed.

**FIGURE 3:**
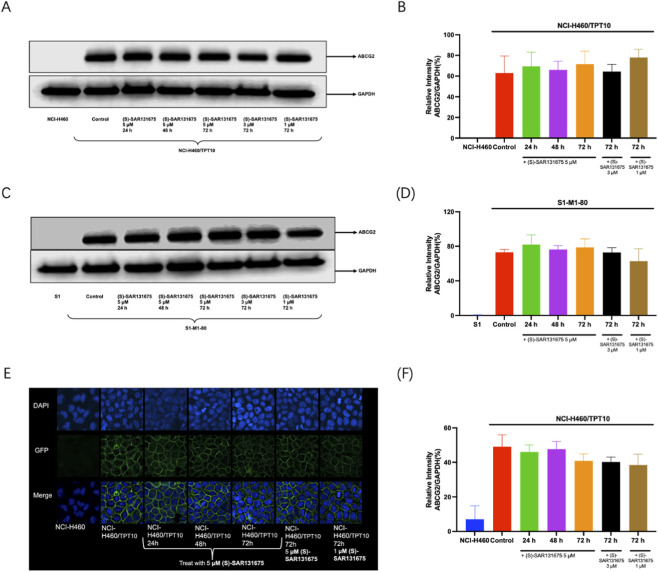
**(A)** Western blotting analysis of ABCG2 expression level in NCI-H460/TPT10 cells after treatment with various concentrations and duration of (S)-SAR131675. **(B)** Quantification of the Western blotting results for ABCG2 protein expression in NCI-H460/TPT10 cells after treatment with different concentrations and durations of (S)-SAR131675. **(C)** Western blotting result of ABCG2 expression level in S1-M1-80 cells after treatment with different concentrations and durations of (S)-SAR131675. **(D)** Quantification of the Western blotting results for ABCG2 expression in S1-M1-80 cells after treatment with various concentrations and durations of (S)-SAR131675. **(E)** Immunofluorescence assay examining ABCG2 localization in NCI-H460/TPT10 cells after treatment with varying concentrations and durations of (S)-SAR131675 (ABCG2 was detected using an Alexa Fluor 488-conjugated secondary antibody [GFP channel]). **(F)** Quantification of immunofluorescence signal intensity for ABCG2 in NCI-H460/TPT10 cells after treatment with various concentrations and durations of (S)-SAR131675. Data are displayed as the mean ± SD of three independent experiments. Nuclei were counterstained with DAPI (blue).

In the Western blotting experiments, NCI-H460/TPT10 and S1-M1-80 cell lines were treated in two modes: time-dependent (0, 24, 48, and 72 h) and concentration-dependent (1, 3, and 5 μM), with the NCI-H460 cell line serving as the negative control. As shown in Figures 3A,C, (S)-SAR131675 did not alter ABCG2 expression in the NCI-H460/TPT10 or S1-M1-80 cells under either condition. Furthermore, the immunofluorescence assay results confirm this; Figures 3E,F show that in NCI-H460/TPT10 cells, ABCG2 remained localized to the cell membrane, and fluorescence intensity did not change significantly. Collectively, these data indicate that the mechanism by which (S)-SAR131675 overcomes ABCG2-mediated MDR does not involve alteration of the expression level or subcellular localization of ABCG2.

### 3.3 (S)-SAR131675 increased the intracellular drug accumulation in the overexpressing ABCG2 cancer cell line

**FIGURE 4:**
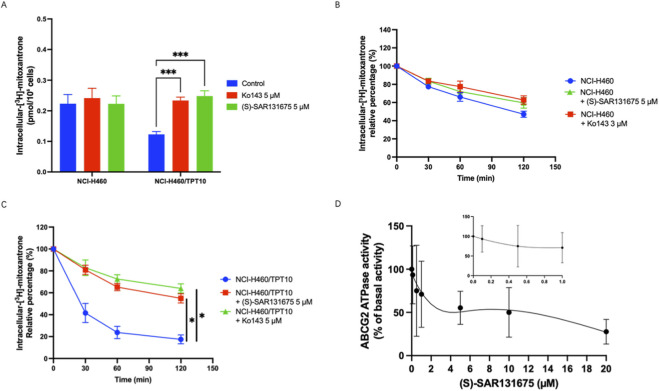
**(A)** Intracellular level of [^3^H]-mitoxantrone in NCI-H460 and NCI-H460/TPT10 cell lines after 2 h incubation with various concentrations of (S)-SAR131675. **(B)** Time-course of intracellular level [^3^H]-mitoxantrone in NCI-H460 with 2 h of treatment with (S)-SAR131675. **(C)** Time course of intracellular level of [3H]-mitoxantrone in NCI-H460/TPT10 with 2 h of treatment with (S)-SAR131675. **(D)** ABCG2 ATPase activity with various concentrations of (S)-SAR131675. Data are displayed as the mean ± SD of three independent experiments. *p < 0.05; **p < 0.01; ***p < 0.001; ****p < 0.0001.

Since the aforementioned experiments ruled out the possibility that the (S)-SAR131675 mechanism is attributable to reduced protein expression or altered protein localization, we subsequently designed another experiment to test the drug’s direct efflux function. First, a [^3^H]-mitoxantrone Accumulation Assay was designed and performed. Figure 4A clearly demonstrates that, compared to NCI-H460 cells, intracellular levels of [^3^H]-mitoxantrone were significantly reduced in the ABCG2-overexpressing NCI-H460/TPT10 cell line, indicating that the accumulation of [^3^H]-mitoxantrone is inhibited by the high expression of ABCG2. Subsequently, we examined whether (S)-SAR131675 could influence this process. In the parental NCI-H460 cell line, treatment with either Ko143 or (S)-SAR131675 did not alter intracellular [^3^H]-mitoxantrone levels. In contrast, in the ABCG2-overexpressing NCI-H460/TPT10 cells, both Ko143 and (S)-SAR131675 significantly increased intracellular levels of [^3^H]-mitoxantrone, suggesting that Ko143 and (S)-SAR131675 partially reverse ABCG2-mediated efflux of [^3^H]-mitoxantrone.

**FIGURE 5:**
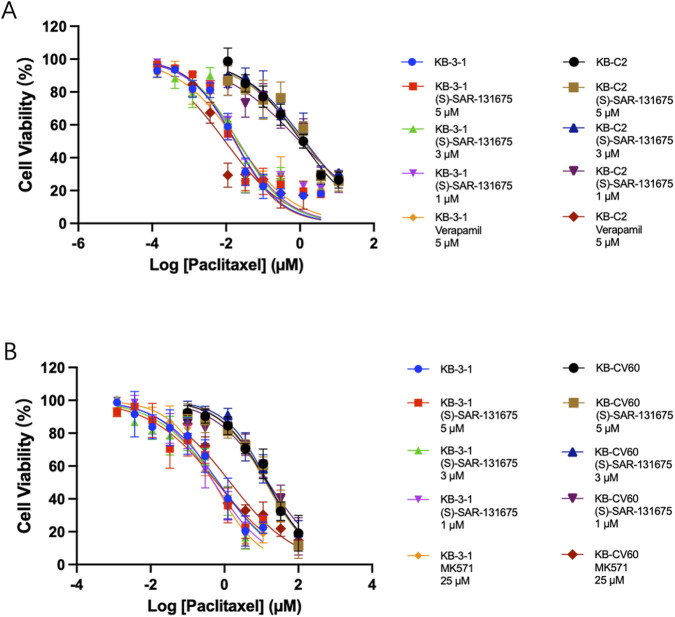
**(A)** Cytotoxicity of paclitaxel in parental KB-3-1 cells and the ABCB1-overexpressing cell line KB-C2 following treatment with paclitaxel alone or in combination with different concentrations of (S)-SAR131675 or verapamil. **(B)** Cytotoxicity of paclitaxel in parental KB-3-1 cells and the ABCC1-overexpressing cell line KB-CV60 following treatment with paclitaxel alone or in combination with different concentrations of (S)-SAR131675 or MK571. Data are presented as mean ± SD from three independent experiments. Data are displayed as the mean ± SD of three independent experiments. *p < 0.05; **p < 0.01; ***p < 0.001; ****p < 0.0001.

### 3.4 (S)-SAR131675 inhibited the efflux function mediated by the ABCG2 transporter in cancer cell lines

To comprehensively assess ABCG2 transporter function, we conducted an efflux assay alongside an accumulation assay. As shown in Figures 4B,C, in the absence of Ko143 or (S)-SAR131675, both the parental NCI-H460 cells and the ABCG2-overexpressing NCI-H460/TPT10 cells exhibited a time-dependent decrease in intracellular [^3^H]-mitoxantrone levels; however, NCI-H460/TPT10 cells exhibited great efflux activity, resulting in further reduced intracellular levels. Furthermore, in the presence of Ko143 or (S)-SAR131675, intracellular [^3^H]-mitoxantrone levels in NCI-H460/TPT10 cells were restored to levels comparable to those observed in NCI-H460 cells, whereas no significant change was detected in NCI-H460 cells upon treatment with either compound. These results demonstrate that (S)-SAR131675 not only affects ABCG2-mediated drug accumulation but also increases intracellular anticancer drug levels, likely through inhibition of ABCG2-mediated efflux.

### 3.5 (S)-SAR131675 suppresses the ATPase activity of ABCG2

ABC transporters, including ABCG2, require ATP hydrolysis to drive substrate efflux. Given that (S)-SAR131675 suppresses ABCG2-mediated efflux, we therefore performed an ATPase assay. This assay was conducted using a series of concentrations of (S)-SAR131675 (0.1–20 μM). As shown in Figure 4D, (S)-SAR131675 significantly suppressed vanadate-sensitive ATPase activity, suggesting that it may inhibit ABCG2-mediated efflux by reducing ATP hydrolysis.

### 3.6 Docking analysis of the binding of (S)-SAR131675 with human ABCG2 homology model

To investigate the potential interaction between (S)-SAR131675 and the ABCG2 transporter, we performed molecular docking analysis. The docking results predicted that (S)-SAR131675 binds within the central transmembrane substrate-binding pocket of the ABCG2 dimer. As shown in Figure 1F, the ligand is positioned deep within the cavity formed by the transmembrane helices of the two monomers. Moreover, as shown in Figure 1G, (S)-SAR131675 is predicted to interact with multiple residues lining the substrate-binding pocket, including Phe432, Phe439, Val546, and Met549, which contribute to the hydrophobic stabilization of the ligand. Additionally, polar interactions with residues such as Asn436 and Thr435 were observed, potentially further stabilizing the ligand’s position within the binding cavity. The docking score of −8.481 kcal/mol suggests that (S)-SAR131675 may favorably interact with the substrate-binding pocket of ABCG2, similar to the docking pattern observed for the positive control Ko143 (−8.708 kcal/mol). These results suggest that (S)-SAR131675 can effectively occupy the substrate-binding cavity of ABCG2, potentially interfering with substrate transport.

## 4 Discussion

With the rapid advancement of medical technology, cancer treatment has also progressed, particularly through the continuous development of chemotherapy drugs and the emergence of targeted therapies and immunotherapies, bringing new hope to cancer patients. However, MDR remains a significant clinical challenge. This challenge continues to drive the development of more effective therapeutic strategies to prevent or overcome MDR (Ge et al., 2025; Chen et al., 2016). Among the different mechanisms of MDR, ABC transporters play a vital role. For example, ABCG2 is widely distributed throughout the human body and plays important physiological roles in maintaining the homeostasis of normal cells; however, in cancer, its overexpression contributes to MDR and adversely affects patient survival (Linton, 2007). Therefore, targeting and suppressing ABCG2 in cancer cells has become a valuable therapeutic strategy, particularly via the combination of anticancer drugs and reversal agents. The limitations of earlier ABCG2 inhibitors have historically hindered their clinical translation. Early compounds, such as fumitremorgin C (FTC), demonstrated potent ABCG2 inhibition but were limited by severe neurotoxicity and poor pharmacological properties (Rabindran et al., 1998; Rabindran et al., 2000). Subsequently developed inhibitors, including novobiocin and GF120918 (elacridar), showed improved potency; however, issues related to selectivity, pharmacokinetic interactions, and off-target effects remained significant concerns (Allen et al., 2002; Kruijtzer et al., 2002). Although third-generation inhibitors, such as Ko143, exhibited greater potency and specificity toward ABCG2, their clinical development has been restricted by limited bioavailability, transporter-related toxicities, and insufficient therapeutic benefit in clinical studies (Kukal et al., 2021; Robey et al., 2004). Additionally, in recent years, increasing evidence has demonstrated that small-molecule targeted agents can enhance the chemosensitivity of cancer cells by modulating ABCG2 activity. Notably, several tyrosine kinase inhibitors (TKIs) have been identified as functional inhibitors of ABCG2, including BCR-ABL inhibitors such as nilotinib, EGFR inhibitors such as erlotinib, and angiogenesis-related kinase inhibitors, including the VEGFR2/3 inhibitor sunitinib and vandetanib, all of which have been reported to increase the intracellular accumulation of chemotherapeutic substrates in several ABC transporters, including ABCG2, ABCC1, and ABCB1 (Zheng et al., 2009; Shukla et al., 2009; Shukla et al., 2012; Shi et al., 2007; Tiwari et al., 2009; Hegedus et al., 2002). VEGFRs are critically involved in tumor angiogenesis, proliferation, and survival. Increasing evidence suggests that activation of VEGFR-related pathways, including PI3K/Akt, MAPK/ERK, STAT3, and HIF-1α signaling, has been reported to regulate ABC transporter expression and function, thereby promoting chemoresistance (Shukla et al., 2006; Chen and Sikic, 2012; Matsuoka and Yashiro, 2014; Mimeault and Batra, 2006). However, the above TKIs, particularly VEGFR2/3 inhibitors sunitinib and vandetanib, have had their clinical translation substantially limited by their broad kinase inhibition profiles and associated dose-limiting toxicities, including hand-foot syndrome, QT prolongation, and hepatotoxicity. These adverse effects are believed to result largely from concurrent inhibition of multiple kinases, including VEGFR1/2, PDGFR, c-KIT, and RET, rather than selective targeting of VEGFR3 (Hu et al., 2009; Mi et al., 2010; Shukla et al., 2009; Shukla et al., 2012; Shi et al., 2007; Tiwari et al., 2009; Zhang et al., 2019). Therefore, identifying a more potent and selective VEGFR inhibitor may help further clarify the relationship between VEGFR signaling and ABCG2-mediated MDR, thereby supporting the development of more effective therapeutic strategies.

(S)-SAR131675 is a selective small-molecule inhibitor of VEGFR3 that has been widely used as a pharmacological tool to study lymphangiogenesis and tumor progression. The kinase selectivity profile of (S)-SAR131675 has been extensively characterized (S)-SAR131675 inhibits VEGFR-3 with an IC_50_ of 23 nM (Ki = 12 nM). In contrast, it shows approximately 10-fold weaker activity against VEGFR-2 (IC_50_ = 235 nM) and >130-fold weaker activity against VEGFR-1 (IC_50_ > 3 μM) (Alam et al., 2012). Importantly, with respect to therapeutic index and clinical translation, the dose-limiting toxicities of multikinase VEGFR inhibitors (e.g., hypertension, proteinuria, and glomerular thrombotic microangiopathy) are mediated primarily by VEGFR-2 inhibition in the systemic vasculature and at the podocyte slit diaphragm, not by VEGFR-3 inhibition (Zhang et al., 2014; Robinson et al., 2010; Eremina et al., 2008). Because VEGFR-3 expression is restricted to lymphatic endothelium and a subset of tumor-associated macrophages, a drug selective for VEGFR-3 would be less likely to induce VEGFR2-mediated cardiovascular and renal toxicities at concentrations sufficient for ABCG2 inhibition. Therefore (S)-SAR131675 may offer a more favorable therapeutic window and potentially provide reduced off-target toxicities. Whether this translates into clinical benefit will depend on confirmation in xenograft and patient-derived models, on whether ABCG2 inhibition occurs at unbound concentrations within the achievable plasma range, and on adequate patient selection for ABCG2-mediated MDR. These considerations highlight challenges that have historically limited the clinical translation of ABCG2 inhibitors. Although VEGFR3 signaling has been implicated in cancer development, the involvement of (S)-SAR131675 in MDR mediated by ABC transporters has not been reported previously. This study provides evidence that (S)-SAR131675 effectively reverses ABCG2-mediated MDR at non-cytotoxic concentrations, and functional reversal studies further demonstrated that (S)-SAR131675 effectively suppressed ABCG2-mediated efflux activity.

Moreover, since ATP hydrolysis is essential for ABCG2 transport activity (S)-SAR131675 suppressed vanadate-sensitive ATPase activity in a concentration-dependent manner, suggesting that (S)-SAR131675 may interfere with the ATP-dependent transport cycle of ABCG2. Moreover, ATPase modulation may occur through substrate-like interaction, competitive inhibition at the substrate-binding pocket, or allosteric effects on transporter conformation. However, current data are insufficient to definitively classify the compound as a transported substrate or as a competitive or noncompetitive inhibitor. Additionally, compared with Ko143, a well-characterized and potent ABCG2 inhibitor (S)-SAR131675 demonstrated a similar overall reversal trend but exhibited distinct ATPase modulation patterns, suggesting that their mechanisms of transporter interaction may not be identical. Therefore, additional mechanistic studies, such as transport competition assays and direct substrate transport analyses, will be required to further characterize the interaction between (S)-SAR131675 and ABCG2.

Although (S)-SAR131675 demonstrated a potent reversal effect of ABCG2-mediated MDR in *vitro* models, the discrepancy between *in vitro* and *in vivo* drug responses remains an important consideration when evaluating its translational potential. Previous studies reported that (S)-SAR131675 was well tolerated in mouse models at a dose of 100 mg/kg/day and significantly inhibited tumor angiogenesis and tumor progression (Alam et al., 2012). In addition, preclinical pharmacokinetic studies demonstrated that oral administration of (S)-SAR131675 achieved measurable systemic exposure in mice, with a reported plasma C_max_ of approximately 11 μM (3.9 μg/mL), indicating that the active concentration range identified in this study is preclinically attainable (Alam et al., 2012). However, because the reported exposure represents total plasma concentration rather than unbound drug concentration, and because clinical pharmacokinetic data remain unavailable, the true translational potential of (S)-SAR131675 as an ABCG2 reversal agent remains uncertain.

Despite these promising results, we acknowledge several limitations in our study. Our research primarily utilized established cancer cell lines and transporter-overexpressing models, which are ideal for investigating specific molecular mechanisms but may not fully capture the complexity of a physiological tumor microenvironment. In addition, whether the MDR reversal effect of (S)-SAR131675 is directly associated with VEGFR3 inhibition or primarily mediated through transporter interactions remains to be clarified. Therefore, future studies using primary tumor models and xenograft systems will be essential for determining the translational applicability of (S)-SAR131675.

## 5 Conclusion

This study employed a series of well-controlled, systematic experiments to demonstrate that (S)-SAR131675 effectively counteracts ABCG2-mediated MDR. Mechanistically, this effect is primarily attributed to the suppression of ABCG2 efflux activity, leading to an enhanced intracellular retention of chemotherapeutic agents and increased drug sensitivity in resistant cancer cells. These results contribute to a deeper understanding of the functional regulation of ABCG2-mediated MDR.

Importantly (S)-SAR131675 demonstrated a robust MDR-reversing effect at preclinically attainable concentrations, underscoring its promise as a chemosensitizer in combination therapy. This therapeutic strategy may improve treatment outcomes in drug-resistant malignancies. However, further investigations, including comprehensive clinical evaluations, are necessary to confirm its efficacy and translational potential in MDR-targeted cancer therapy.

## Data Availability

The raw data supporting the conclusions of this article will be made available by the authors, without undue reservation.
